# A Technical System for the Large-Scale Application of Metabolites From *Paecilomyces variotii* SJ1 in Agriculture

**DOI:** 10.3389/fbioe.2021.671879

**Published:** 2021-05-12

**Authors:** Qingbin Wang, Chune Peng, Liran Shi, Zhiguang Liu, Dafa Zhou, Hui Meng, Hongling Zhao, Fuchuan Li, Min Zhang

**Affiliations:** ^1^National Engineering Laboratory for Efficient Utilization of Soil and Fertilizer Resources, College of Resources and Environment, National Engineering and Technology Research Center for Slow and Controlled Release Fertilizers, Shandong Agricultural University, Tai’an, China; ^2^Shandong Pengbo Biotechnology Co., Ltd., Tai’an, China; ^3^State Key Laboratory of Crop Biology, College of Life Sciences, Shandong Agricultural University, Tai’an, China; ^4^Shandong Key Laboratory of Carbohydrate Chemistry and Glycobiology, National Glycoengineering Research Center, Shandong University, Qingdao, China

**Keywords:** endophytes, metabolites, *Paecilomyces variotii* SJ1, large-scale application, agriculture

## Abstract

Compared with endophytes, metabolites from endophytes (MEs) have great potential in agriculture. However, a technique for industrializing the production of MEs is still scarce. Moreover, the establishment of effective methods for evaluating the quality of MEs is hampered by the fact that some compounds with beneficial effects on crops have not been clearly identified. Herein, a system was established for the production, quality control and application of MEs by using the extract from *Paecilomyces variotii* SJ1 (ZNC). First, the extraction conditions of ZNC were optimized through response surface methodology, after which each batch (500 L) met the consumption requirements of crops in 7,467 hectares. Then, chromatographic fingerprinting and enzyme-linked immunosorbent assay were applied to evaluate the similarity and specificity of unknown effective components in ZNC, ensuring a similarity of more than 90% and a quantitative accuracy of greater than 99.9% for the products from different batches. Finally, the bioactivity of industrially produced ZNC was evaluated in the field, and it significantly increased the potato yields by 4.4–10.8%. Overall, we have established a practical technical system for the large-scale application of ZNC in agriculture.

## Introduction

Symbiotic relationships between endophytic microbes and plants are ancient and conserved in most land plants (Gundel et al., 2013). As a result of these long-held associations, host plants have devised genetic systems allowing for the transfer of information between them and their endophytic microbes (Stierle et al., 1993; Wei and Jousset, 2017). Obviously, this would provide a rapid and reliable mechanism for them to deal with everchanging environmental conditions (Rillig et al., 2015). Therefore, plants infected with endophytes have the advantages of rapid growth (Koprivova et al., 2019), stress resistance (de Vries et al., 2020) and disease resistance (Carrion et al., 2019), which gives them more survival advantages than plants not infected by endophytes (Wei and Jousset, 2017). However, once endophytes are applied to fields, they fight against antagonistic factors from soil and other microbial colonies to survive (Bolwerk et al., 2003; Dolfing and Feng, 2015). In addition, the immune killing of the invading microorganisms caused by the target host and the competition of the epiphytic microorganisms in the niche are not conducive to the formation of new symbiotic relationships (Liu et al., 2020). Moreover, excessively competitive endophytes greatly challenge the safety of the ecological environment because beneficial and pathogenic microbes share physiological features and evolutionary proximity to an extent that the manifestation of a pathogenic phenotype may depend on small differences in the microbe and sometimes even on the host (Rodriguez et al., 2019). Therefore, despite widespread claims of the efficacy of inoculation with plant growth-promoting endophytes under laboratory conditions, we were unable to find studies demonstrating attribution of the beneficial effect to a specific selected trait, and there is limited evidence of inoculation success and subsequent benefits for plant growth in fields (de Vries et al., 2020; Dini-Andreote, 2020; Liu et al., 2020).

Accumulating evidence suggests that metabolites from endophytes (MEs), such as C-terminally encoded peptides, microRNAs, auxin, and strigolactones (Oldroyd and Leyser, 2020), have a pivotal role in facilitating the direct communication between plants and microbes via signaling molecules such as phytohormones and thus regulate shoot growth or control the levels of nutrition importers (Khare et al., 2018; Acuña-Rodríguez et al., 2020). Interestingly, most of the MEs described act as precursors of immune phytohormones; for example, phenylalanine is a precursor in salicylic acid biosynthesis and involved in other stress responses (de Vries et al., 2020). Moreover, MEs are more stable and eco-free than ever-changing endophytes (Schenk et al., 2012; Oldroyd and Leyser, 2020), which might result in new opportunities to increase plant performance with particular benefits for crop production (White et al., 2019). However, despite the new technologies through which MEs are extracted in the laboratory and the subsequent use of MEs to increase plant growth and fitness, a technique for industrializing the production of MEs is still scarce (Qin et al., 2018). Moreover, the establishment of effective methods for monitoring and evaluating the quality of ME is hampered by the fact that some compounds with beneficial effects on crops have not been clearly identified (Brader et al., 2014).

The results of previous studies have shown that extract from *Paecilomyces variotii* SJ1 (ZNC) can promote the growth of *Arabidopsis* roots and increase the utilization of nitrogen and phosphorus in *Arabidopsis* at concentrations of 1–10 ng mL^–1^ in the laboratory (Lu et al., 2019). Furthermore, the utilization efficiency of nitrogen or phosphorus in rice, corn and wheat in the field was improved when ZNC was applied at a rate of 375 mg⋅ha^–1^, which subsequently increased the yield of the crop (Chen et al., 2020; Wang et al., 2020). However, laboratory-produced ZNC not only cannot meet its large-scale application requirements but also is inconsistent between different batches due to the ineffective detection and evaluation of active ingredients in ZNC.

In this study, a technical system for industrial production and evaluation of MEs featuring the function of endophytes was designed and used to produce ZNC. First, ultrasonic extraction (UAE) and response surface methodology were employed to industrialize the highly efficient, low-harm production of ZNC. Furthermore, the similarity evaluation system of traditional Chinese medicine chromatographic fingerprinting (SES-TCMCF), multiple antibody enzyme-linked immunosorbent assay (MA-ELISA) and field experiments were combined to guarantee the consistent quality and high activity of ZNC. The innovative technology for popular application of MEs was successful, and it is expected to improve agricultural production by enabling the use of less fertilizer and pesticide.

## Materials and Methods

### Materials

Mycelium of SJ1 (CGMCC No. 10114) was provided by Shandong Pengbo Biotechnology Co., Ltd. A bicinchoninic acid protein quantification kit, an Ezup column fungal genomic DNA extraction kit, blood vessel collection equipment, a goat anti-rabbit horseradish peroxidase marker, tetramethylbenzidine, 10 mM phosphate buffer solution (PBS) at pH 7.2–7.4, 0.05% tween-20 in PBS and bovine serum albumin were purchased from Beijing Solarbio Science and Technology Co., Ltd., China. Freund’s complete adjuvant, Freund’s incomplete adjuvant, glucose standard (purity ≥ 99%), cholesterol standard (C_27_H_46_O, purity ≥ 99%), and L-glutamate standard (purity ≥ 99%) were purchased from Sigma-Aldrich Co. Ltd., United States. Methanol, acetic acid, acetonitrile and ammonium acetate were high-performance liquid chromatography (HPLC) grade and purchased from Thermo Fisher Scientific, China. Industrial alcohol (purity ≥ 95%) were purchased from Jinan Hongzheng Chemical Co., Ltd, China. Hydrochloric acid, sulfuric acid, phenol, petroleum ether, phosphoric acid, potassium iodide, iodine, ammonium ferric sulfate, potassium hydroxide, sodium hydroxide, and all other chemicals and reagents were analytical grade and purchased from Sinopharm, China.

### Optimization of Ultrasonic Extraction Parameters for Extracting ZNC

Mycelium from endophytic *Paecilomyces variotii* SJ1 (SJ1) obtained by three-stage liquid fermentation was filtered in a plate and frame filter press (XMYG30, Yuzhou Dachang Filtration Equipment Co., Ltd., China) with filter cloth (300 mesh) by an air compressor at a pressure of 0.2 MPa. Subsequently, mycelium was obtained by pressure filtration at 0.4 MPa and then mixed with the same volume of 95% alcohol. The mixture was sealed for 2 weeks for extraction of ZNC.

Response surface methodology (RSM) was used to determine ultrasonic extraction parameters under a central composite design (CCD) model. CCD was used to produce a design matrix of a four-factor, five-level rotatable model containing 30 experimental runs with six replicates at the central point, which is given in Table 1. The four experimental factors included the proportion of material (X_1_: 0, 10, 20, 30, and 40%, by mass concentration), ethanol concentration (X_2_: 0, 10, 20, 30, and 40%, volume concentration), extraction time (X_3_: 0, 30, 60, 90, and 120 min) and power (X_4_: 0, 1.5, 3.0, 4.5, and 6.0 kW) corresponding to energy densities (X_4_: 0, 3, 6, 9, and 12 J⋅L^–1^) in a second. The yield of ZNC (Y) was chosen as the response (dependent) variable. The order of the experiments was randomized to minimize selection bias and the effect of unexplained variability on the observed responses due to systematic errors.

**TABLE 1:** Design and result of central composite design (CCD).

X_1_: Proportion of material (%)	X_2_: Concentration of alcohol (%)	X_3_: Extraction time (min)	X_4_: Power (kW)	Y: Concentration of ZNC (mg⋅mL^–^^1^)
20	0	60	3.0	5.75
20	40	60	3.0	7.27
20	20	60	3.0	7.06
20	20	0	3.0	3.86
10	10	90	4.5	2.42
30	10	90	1.5	8.93
20	20	60	3.0	6.98
30	10	30	4.5	8.02
20	20	60	3.0	5.95
20	20	60	0.0	7.2
40	20	60	3.0	12.78
20	20	60	3.0	7.2
10	30	30	1.5	2.84
30	30	90	1.5	9.68
10	30	30	4.5	3.34
30	30	90	4.5	10.87
10	10	90	1.5	2.48
30	10	30	1.5	9.04
20	20	120	3.0	5.86
30	30	30	4.5	11.43
30	30	30	1.5	9.68
10	10	30	1.5	2.19
10	30	90	1.5	3.36
10	30	90	4.5	4.54
20	20	60	3	6
20	20	60	6	6.47
0	20	60	3	0
10	10	30	4.5	2.3
20	20	60	3	6.04
30	10	90	4.5	7.79

The corresponding materials, alcohol and water were injected into a 500-liter (L) UAE tank (FGC-TQ/7/5/6, Jining Jinbaite Biological Machinery Co., LTD., China) according to the design matrix. UAE was performed at 25°C with a frequency of 20.0 kHz and a stirring speed of 100 × g min^–1^. Then, the mycelium residue was removed by a filter press, and the extract was named ZNC as described previously (Lu et al., 2019; Chen et al., 2020; Peng et al., 2020). The extract (10 mL) was then dried to a constant weight at 40°C in a vacuum rotary concentrator at 1,200 × g min^–1^. Furthermore, the optimal extraction conditions were determined by the “desirability” algorithm (Ghosh et al., 2012) using ultrasonic extraction data. The “goal category” of the experimental variables was set as “in the range”, whereas that of response variables was set as “maximize”. The UAE parameters yielding the highest desirability were selected as the optimal extraction conditions.

### Composition and Molecular Weights of ZNC Components

The content of organic matter in ZNC was measured using a TOC-5000A (Shimadzu, Japan) according to carbon content (Seifert et al., 2016). The total saccharide content in ZNC was determined by the phenol sulfate method. The total protein content in ZNC was quantified with a bicinchoninic acid protein quantitation kit. Total nucleic acids in ZNC were extracted by an Ezup Column Genome DNA Extraction Kit and measured by a Q5000 UV-Vis Spectrophotometer (Quawel, United States). Total amino acid content was measured by ninhydrin assay. Total lipid content was measured based on the National Standard of China GB 5009.128-2016.

To determine the molecular weight of ZNC components, 100 μL of ZNC (10 mg mL^–1^) was loaded onto a pre-equilibrated Superdex peptide 10/300 GL column (GE Healthcare, United States), which was calibrated using size-defined hyaluronic acids, glucuronic acid and glutamic acid at 35°C with HPLC (Peng et al., 2018). Then, the eluted fractions were monitored at 210 nm with a UV detector SPD-10A (Shimadzu, Japan). The mobile phase was 0.2 M ammonium hydrogen carbonate at a flow rate of 0.4 mL min^–1^. Online monitoring and data analysis were performed using LC solution version 1.25 software.

An analysis of the molecular weight distribution was performed on a Xevo TQD mass spectrometer (MS) (Waters, United States) with an electrospray ion source (ESI) at 40°C. The mobile phase consisted of 0.1% acetic acid in water (A) and acetonitrile (B) under positive-ion mode and water (A) and acetonitrile (B) under negative-ion mode. The elution gradient flow rate was maintained at 200 μL⋅min^–1^ using 50% A. The MS analysis was performed with the following instrument parameters: capillary voltage, 3,600 V (ESI+)/3,000 V (ESI−); desolvation temperature, 360°C; source temperature, 120°C; desolvation gas flow rate, 600 L h^–1^; cone gas flow rate, 50 L h^–1^. A full scan covering the range from 50 to 1,000 m/z was chosen.

### Establishment of Evaluation Method for Similarity of ZNC

According to the composition and molecular weights of ZNC components, an amino chromatographic column YMC–Pack Polyamine-II 260 mm × 4.6 mm, 5 μm (YMC, Japan) was used as the stationary phase at 40 °C, while water (A) and acetonitrile (B) were selected as the mobile phases. The separation was optimized through different elution time programs at a flow rate of 1 mL min^–1^ with an LC-20A system (Shimadzu, Japan) and monitored by measuring the absorbance at 210 nm. Furthermore, a fingerprint similarity evaluation of ZNC was established using SES-TCMCF (2012.130723), which could evaluate the quality of ZNC among different batches and the factors in the ZNC extraction process that affect this quality.

### Establishment of Method for Evaluating the Specificity of ZNC

The specificity of ZNC may be detected by MA-ELISA based on their immunogenicity and antigenicity. Blood (5 mL) was collected from the venous vessels of six male New Zealand rabbits (6 weeks old) and saved as a blank control. After 1 week, 1 mg of ZNC in 1 mL of PBS was mixed with Freund’s complete adjuvant at a volume ratio of 1:1 until they were emulsified thoroughly using a three-way valve with two syringes, and the mixed emulsion was injected into the subcutaneous tissue of a rabbit for initial immunization. A total of eight injection sites were distributed on both sides of the spine, and each site was injected with 200 μL. Thereafter, the rabbits were immunized again with another emulsifier containing 0.5 mg of ZNC and 1 mL of Freund’s incomplete adjuvant (volume ratio 1:1) every 2 weeks for three times in total to maximize the production of specific antibodies circulating in the blood (titer). Then, blood (5 mL) was collected from the rabbit’s auricular vein before every immunization. When the titer of serum reached 10^5^, all the blood of rabbits was collected. The serum titer was determined by indirect ELISA using goat anti-rabbit antibody labeled with horseradish peroxidase as the secondary antibody (Natarajan and Remick, 2008). Then, the concentration of antigens in ELISA was optimized using dilution factors of ZNC (10 mg mL^–1^) of 5,000, 10,000, 20,000, 50,000, 100,000, 200,000, 500,000, and 1,000,000 when the dilution factor of the serum was 1,000. Finally, the dosages of antigen and antibody were further optimized for the qualitative and quantitative evaluation of ZNC.

### Testing the Biological Activity of ZNC on Field Potatoes

A field experiment was conducted during one potato growing season (March–September 2019) using “Favorita” at Huamawan township, Tai’an City, Shandong Province, China (36°09′15′N, 117°09′02′E). The physical and chemical properties of the soil (0–20 cm) before planting were as follows: pH 8.19 (soil to water ratio 1:2.5); organic matter content, 15.13 g kg^–1^; alkaline hydrolysis nitrogen (N), 50.39 mg⋅kg^–1^; available phosphorus (P), 11.55 mg⋅kg^–1^; and available potassium, 110 mg⋅kg^–1^. Common urea (46% N), superphosphate (16% P_2_O_5_) and potassium sulfate (50% K_2_O) were freely supplied by Kingenta Ecological Engineering Group Co., Ltd. (China). A randomized complete block design was used with six treatments: U_2/3_Z_0_ (100 kg N ha^–1^); U_2/3_Z_1_ (100 kg N combined with ZNC ha^–1^); U_1_Z_0_ (150 kg N ha^–1^); U_1_Z_1_ (150 kg N combined with ZNC ha^–1^); U_4/3_Z_0_ (200 kg N ha^–1^); U_4/3_Z_1_ (200 kg N combined with ZNC ha^–1^); and ZNC with urea at a rate of 375 mg ha^–1^ mixed in a rotating drum. At the same time, the diluents of ZNC were added at the seedling stage (29th day) and tuber germinating stage (45th day), and the concentration of ZNC diluent was 10 ng mL^–1^ based on 375 mg⋅ha^–1^ every time, while an equal amount of clean water was added for the control treatment. All treatments included 90 kg ha^–1^ P_2_O_5_ and 210 kg⋅ha^–1^ K_2_O, and all fertilizers were applied once at a depth of 15 cm in the center of the ridge. Each treatment was conducted in triplicate. The plot area was 3 m × 5 m = 15 m^2^. Two rows of plants were planted 20 cm apart on the same ridge with a plant spacing of 25 cm, and the width of the ridge was 80 cm. The actual density was approximately 125,000 plants⋅ha^–1^.

Samples (10 plants) were obtained at the mature stage (101 days). Plant samples except for those some potato tubers were desiccated at 105°C for 30 min and oven-dried at 70°C in a forced air circulation oven until a constant weight was reached (Silva et al., 2013). The total N concentration of the plants was determined by H_2_SO_4_–H_2_O_2_ digestion and a micro-Kjeldahl procedure (Douglas, et al., 1980). Soluble protein content in fresh potato tubers was determined using a colorimetric method with Coomassie brilliant blue G-250. Vitamin C content in fresh potato tubers was probed using 2,6-dichloroindophenol titration. The determination of reducing sugar content was carried out by a 3,5-dinitrosalicylic acid colorimetric method, and the starch content of fresh potato tubers was measured by a gravity method (Zebarth and Rosen, 2007).

### Statistical Analysis

The optimization of the UAE conditions was performed using the trial version of Design Expert 12.0.3.0 (Stat-Ease Inc., MN, United States). The model was subjected to an analysis of variance (ANOVA). The quality of the polynomial model equation was judged statistically by the coefficient of determination *R*^2^, and its statistical significance was determined by an *F*-test. The significance of the regression coefficients was tested by a *t*-test. Microsoft Excel 2007 was adopted for data processing, and Origin 8.5.1 was used to draw figures. Data were analyzed with the Statistical Analysis System package version 8.1 (2006, SAS Institute, Cary, NC, United States). ANOVA with Duncan’s multiple range tests was adopted to assess the differences among the means of three replicates from each treatment at a critical *P* value of 0.05.

## Results

**FIGURE 1:**
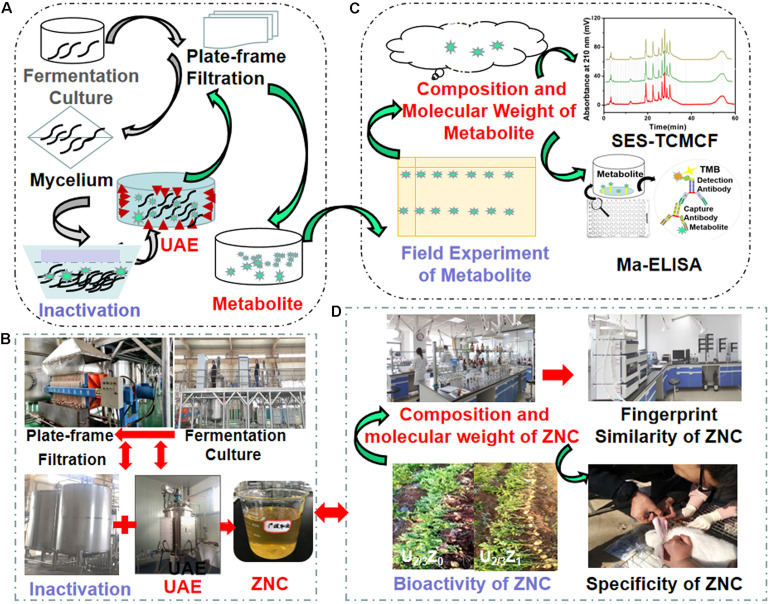
Schematic illustration and realistic picture of extract from *Paecilomyces variotii* SJ1 (ZNC). **(A,B)** Schematic illustration of industrial extraction and evaluation of ZNC, respectively; and **(C,D)** realistic picture of extraction and evaluation of ZNC, respectively.

According to the process in Figure 1, the industrial extraction and evaluation of ZNC were performed.

### Optimization of ZNC Extraction by RSM

To optimize the major UAE conditions that affecting the yield of ZNC, RSM was employed under CCD mode. The concentrations of ZNC used in the design matrix are shown in Table 1, and the model that can be used to navigate the design space was further analyzed using Design Expert 12.0.3.0 software (trial version). The model was as follows:

$$\begin{array}{l} Y=-1.50167+0.345542X_{1}-0.071958X_{2}+0.071264X_{3}\\ \;-0.650833X_{4},+0.001994X_{1}X_{2}-0.000631X_{1}X_{3}\\ \;-0.003958X_{1}X_{4}+0.000227X_{2}X_{3}+0.028042X_{2}X_{4}\\ \;-0.000236X_{3}X_{4}-0.000316X_{1}^{2}+0.000016X_{2}^{2}\\ \;-0.000460X_{3}^{2}+0.035417X_{4}^{2} \end{array}$$

where *Y* is the predicted ZNC yield, *X*_1_ is the content of the material, *X*_2_ is the alcohol concentration, *X*_3_ is the extraction time, and *X*_4_ is the extraction power.

**FIGURE 2:**
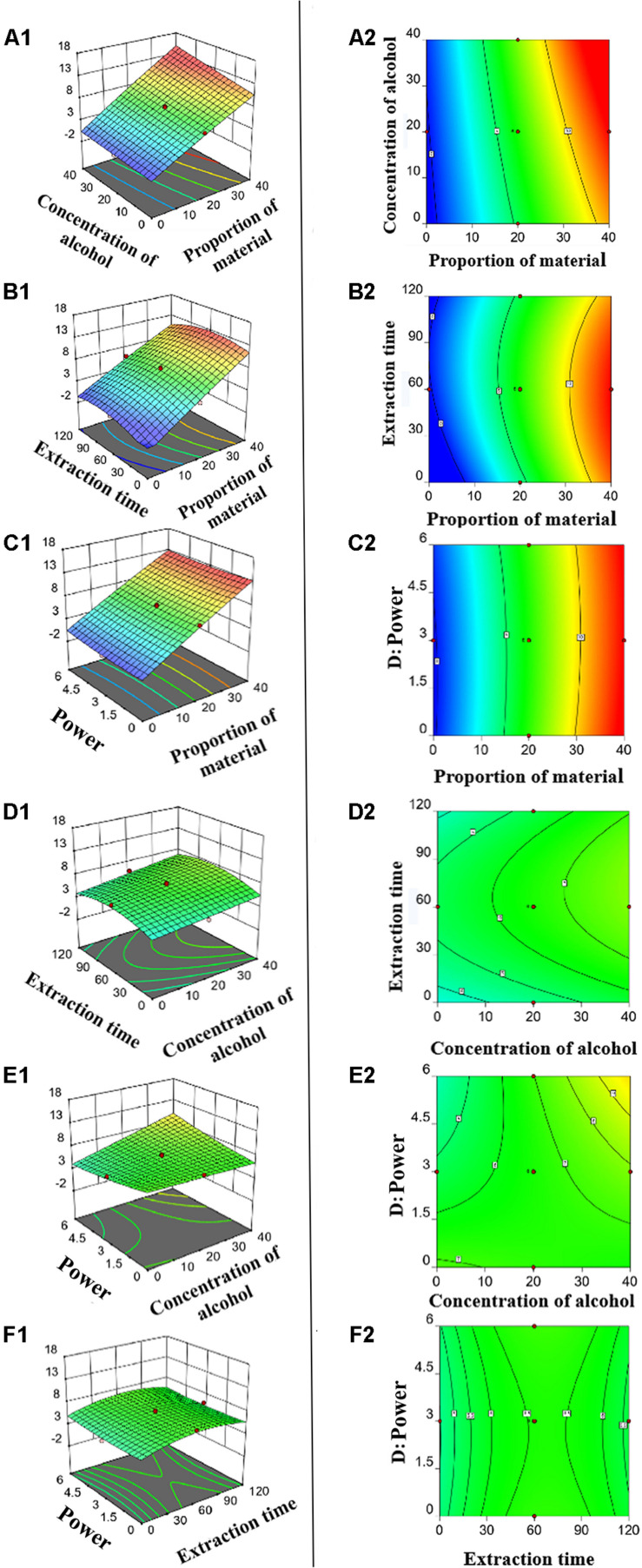
Three-dimensional response plots **(A1–F1)** and contour plots **(A2–F2)** for optimized process parameters**. (A1,A2)** Proportion of material versus concentration of alcohol. **(B1,B2)** Proportion of material versus time. **(C1,C2)** Proportion of material versus power. **(D1,D2)** Concentration of alcohol versus time. **(E1,E2)** concentration of alcohol versus power. **(F1,F2)** time versus power.

According to *t-*tests and *P-*values, X_1_ (*P* < 0.0001), X_2_ (*P* < 0.0001), X_2_X_4_ (*P* < 0.0131), and ${\text{X}}_{3}^{2}$ (*P* < 0.0025) had significant effects on the concentration of ZNC (Table 2); in particular, X_2_ exerted this influence not only by itself but also by interacting with X_4_. To better present the effects of variables on ZNC production, the predicted model was visualized in 3D response surface graphs and contours (Figure 2). Moreover, the maximum concentration of ZNC was 16.8 mg mL^–1^, which occurred when X_1_ was 40.0%, X_2_ was 40.0%, X_3_ was 60 min, and X_4_ was 6.0 kW or 12 J L^–1^. The correlation coefficient *R*^2^ of this model was greater than 0.9806, which indicated that 98.06% of the sample variation was attributed to the variables and that the total variance that could not be explained by the model was less than 2% (Ghosh and Hallenbeck, 2010; Ghosh et al., 2012). The lack of fit (*P* = 0.536) was not significant, suggesting that the model is adequate for prediction within the range of variables employed (Chen et al., 2009). The adjusted determination coefficient (*R*^2^ = 96.25%) and adeq precision (30.574) (Table 2) were also satisfactory to confirm the prediction accuracy of the model. In addition, the model was extremely significant, with a *P* value of < 0.0001 at a confidence level of 95%.

**TABLE 2:** Analysis of variances (ANOVA) for the quadratic model.

Source	Sum of squares	d*f*	Mean square	*F*-value	*P*-value	
**Model**	271.26	14	19.38	54.24	<0.0001	Significant
X_1_-Proportion of material	199.02	1	199.02	557.10	<0.0001	
X_2_-Concentration of alcohol	12.97	1	12.97	36.31	<0.0001	
X_3_-Extraction time	0.8836	1	0.8836	2.47	0.1366	
X_4_-Power	0.1778	1	0.1778	0.4977	0.4913	
X_1_X_2_	0.6360	1	0.6360	1.78	0.2020	
X_1_X_3_	0.5738	1	0.5738	1.61	0.2244	
X_1_X_4_	0.0564	1	0.0564	0.1579	0.6967	
X_2_X_3_	0.0743	1	0.0743	0.2079	0.6550	
X_2_X_4_	2.83	1	2.83	7.92	0.0131	
X_3_X_4_	0.0018	1	0.0018	0.0051	0.9443	
${\text{X}}_{1}^{2}$	0.0273	1	0.0273	0.0765	0.7859	
${\text{X}}_{2}^{2}$	0.0001	1	0.0001	0.0002	0.9893	
${\text{X}}_{3}^{2}$	4.70	1	4.70	13.16	0.0025	
${\text{X}}_{4}^{2}$	0.1742	1	0.1742	0.4875	0.4957	
**Residual**	5.36	15	0.3572			
**Lack of Fit**	3.57	10	0.3569	0.9974	0.5362	Not significant
Pure error	1.79	5	0.3579			
**Cor total**	276.61	29				
**Fit of the model**	**Adeq precision**		**Adjusted *R*^2^**		**Predicted *R*^2^**	
	30.574		0.9625		0.9160	

### Composition and Molecular Weights of ZNC Components

**FIGURE 3:**
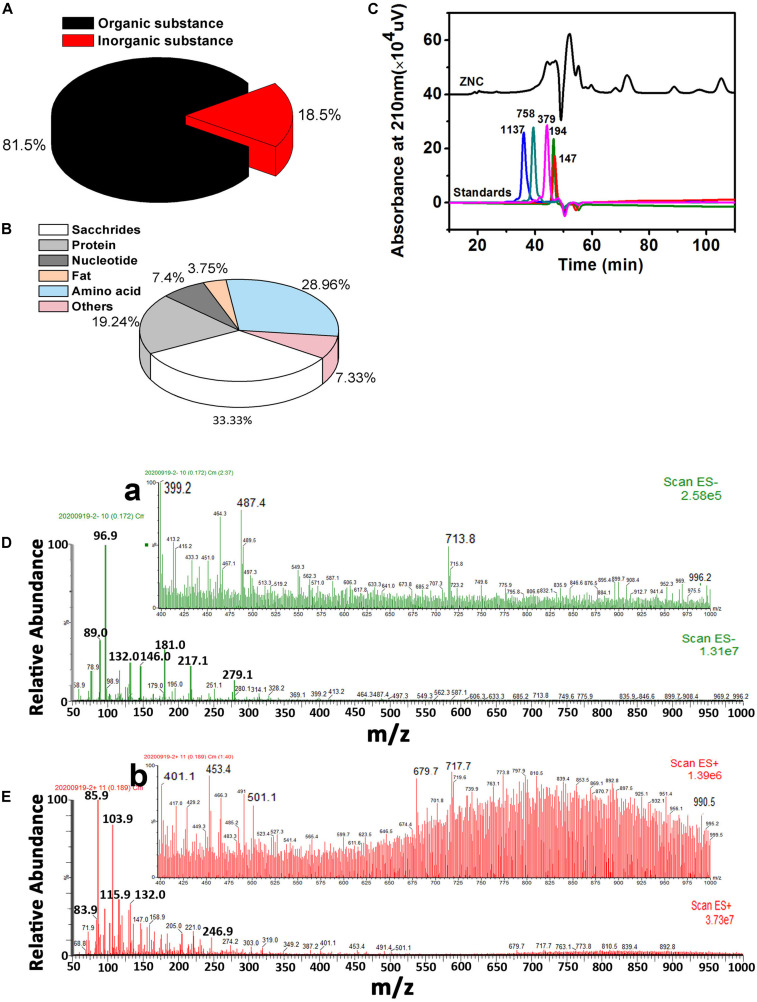
Main components and molecular weight distribution of ZNC. **(A)** Organic and inorganic contents in ZNC. **(B)** Main component in organic matter of ZNC. **(C)** Average molecular weight of ZNC determined by size exclusion chromatography, and the numbers above the characteristic peaks indicate the molecular weight of the molecule with that retention time. **(D,E)** Molecular weight distribution of the ZNC as determined by LC-ESI-MS under positive-ion mode and negative-ion mode, respectively. **(a,b)** enhanced view of D and E in the mass-charge ratio range of 400–1,000.

In ZNC, the organic matter content was 81.5% (Figure 3A). The saccharide content was 33.33%, the protein content was 19.24%, the amino acid content was 28.96%, the nucleoside content was 7.4% and the lipid content was 3.75% in the organic matter (Figure 3B). The average molecular weight of ZNC was determined by HPLC, and the molecular weight distribution of ZNC was further characterized by LC-ESI-MS under full scanning mode. The average molecular weight of ZNC was less than 758 Da (Figure 3C), and the molecular weights were mainly distributed in the range of 70–500 Da (Figures 3D,E). However, a small number of compounds were detected in the range of 600–1,000 Da under negative-ion mode (Figures 3a,b).

### Evaluation Method of Fingerprint Similarity for ZNC

**FIGURE 4:**
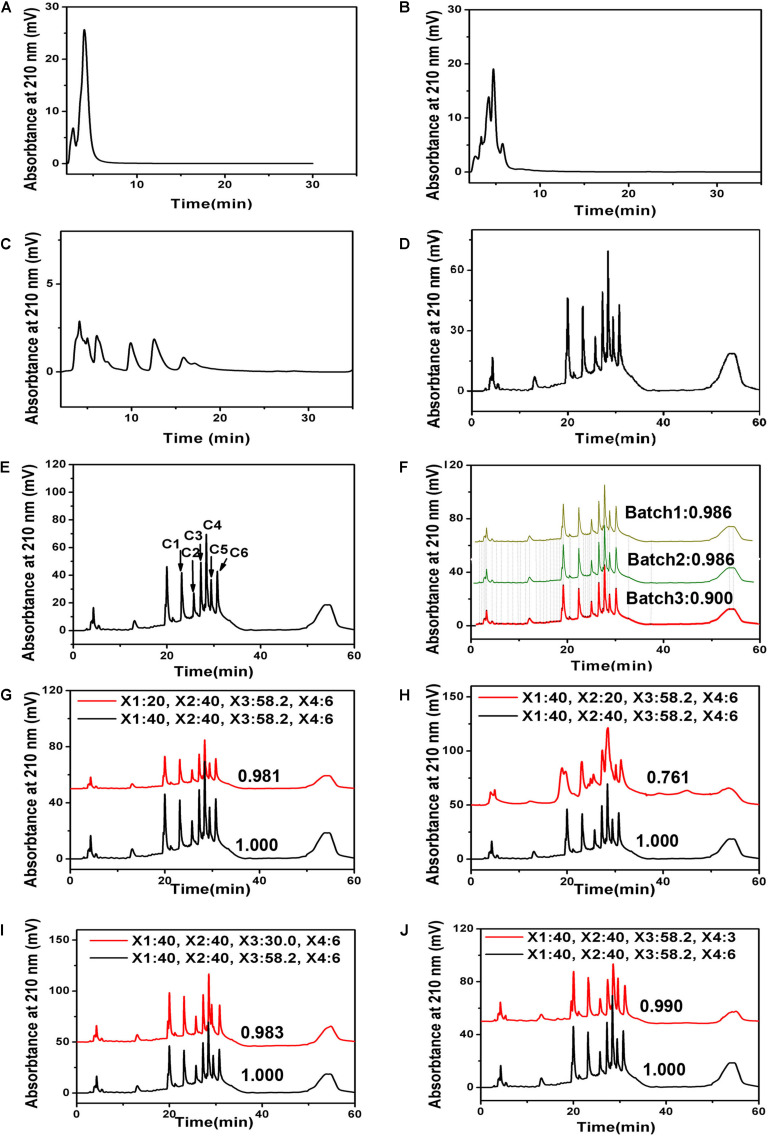
Similarity evaluation system of traditional Chinese medicine chromatographic fingerprinting (SES-TCMCF) of ZNC. High-performance liquid chromatography (HPLC) of ZNC with different elution conditions. **(A)** 30% methanol, **(B)** 60% methanol, **(C)** 90% methanol, **(D)** 95% methanol for 5 min followed by linear gradients from 95 to 90% methanol from 5 to 10 min, from 90 to 60% methanol over 10–20 min, and from 60 to 0% methanol over 20–28 min; finally, 0% methanol was maintained from 28 to 43 min, **(E)** characteristic peaks used for quantitative analysis of ZNC; **(F)** similarity and HPLC among different batches of ZNC using SES-TCMCF; **(G–J)** similarity and chromatograms for comparing extractions with different process parameters (red line) and extractions with optimal process parameters (batch1).

To evaluate the quality of ZNC containing unknown components, the professional software SES-TCMCF was used to measure the similarity among different batches of ZNC based on the numbers, abundances and retention times of characteristic peaks in their chromatograms. First, under different elution gradients (Figures 4A–D), the retention times and separation degrees of characteristic peaks were optimized to obtain a better chromatogram that could be used in SES-TCMCF (Figure 4F). In addition, there were six characteristic peaks (Figure 4E) whose correlation coefficients were greater than 0.99 between the integral area and content of the samples (Liang et al., 2010), and they were selected for calculations of the ZNC content (Jiao et al., 2015; Zhang et al., 2019). A validation experiment was performed in triplicate tests under the optimized UAE conditions. The similarity was greater than 0.90 (Figure 4F), the RSD of the retention time was less than 0.06% (*n* = 3), and the RSD of the peak area was less than 1.42% (*n* = 3) (Table 3) among different batches of ZNC, suggesting that the different batches of ZNC were similar (Gao et al., 2013). Finally, the chromatograms and similarities of ZNC between extractions with different process parameters (red line) and optimal process parameters (X_1_: 40%, X_2_: 40%, X_3_: 58.2 min, and X_4_: 6 kW) (black line) were compared to investigate the factors that affected the ZNC extraction (Figures 4G–J) (Jiao et al., 2015; Yang et al., 2017; Zhang et al., 2019). The chromatogram of ZNC extracted using the process parameters X_1_ 40%, X_2_ 20%, X_3_ 58.2 min, and X_4_ 6 kW (Figure 4H) had obvious differences compared with that of ZNC extracted using the optimal process parameters, suggesting that X_2_ is the main factor that affects the composition of metabolites during the extraction. Moreover, X_1_, X_3_, and X_4_ variations had little effect on the chromatograms and similarities of ZNC samples (Figures 4G,I,J).

**TABLE 3:** Analysis of variances of characteristic peak.

Treatment	C1	C2	C3	C4	C5	C6
*R*^2^ of linear correlation	0.997	0.997	0.990	0.996	0.991	0.9984
RSD of retention time	0.02	0.00	0.02	0.02	0.06	0.02
RSD of peak area	0.36	1.42	0.75	0.64	0.49	0.39

### Evaluation Method of Specificity for ZNC

**FIGURE 5:**
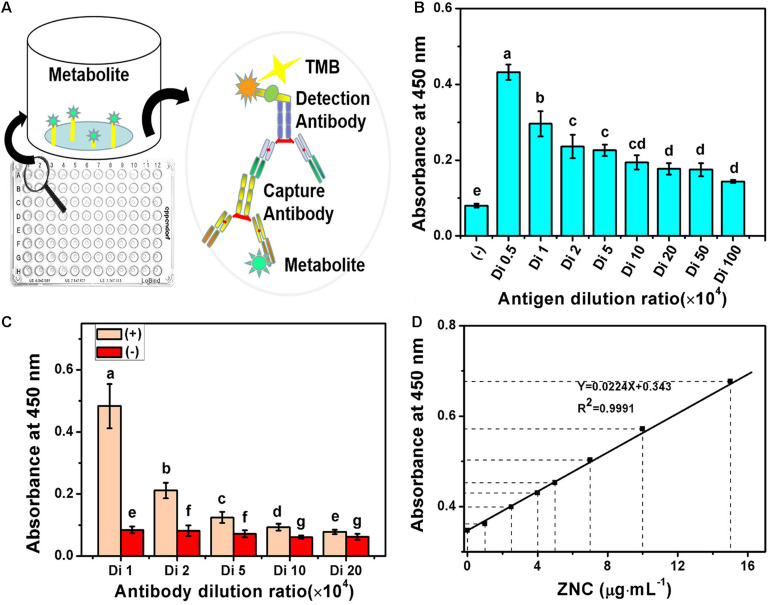
Multiple antibody enzyme-linked immunosorbent assay (MA-ELISA) for ZNC established using indirect-ELISA. **(A)** Schematic illustration, **(B)** antigen sensitivity, **(C)** antibody titer, and **(D)** linear correlation coefficient of ZNC using MA-ELISA. *Note:* Means on top of each column followed by the same letter were not significantly different at the 0.05 level based on one-way analysis of variances (ANOVAs) followed by Duncan’s multiple range test.

To detect and quantify ZNC components more accurately, MA-ELISA, a method complementary to SES-TCMCF, was further employed. Various immune factors in ZNC stimulate the immune response of New Zealand white rabbits and subsequently produce corresponding antibodies and memory cells (Desmet et al., 2013). After the immune response was strengthened three times, the antibodies in rabbits exponentially grew (van de Gaer et al., 2020) and were obtained from whole blood. Then, ZNC was detected by indirect ELISA as depicted in Figure 5A. The results showed that the titer of ZNC was ≧10^6^ when the antibody dilution was 1,000-fold (Figure 5B) and that the titer of the antibody was ≧10^5^ when the ZNC was diluted 1,000-fold (Figure 5C), which could meet the requirements of ELISA (Lequin, 2005). The optimized conditions for qualitative and quantitative evaluation of ZNC were that the antigen was diluted 1,000–10,000-fold when the antibody was diluted 5,000-fold, and their correlation coefficient *R*^2^ was 0.9991 (Figure 5D).

### Bioactivity of ZNC in Fields

**FIGURE 6:**
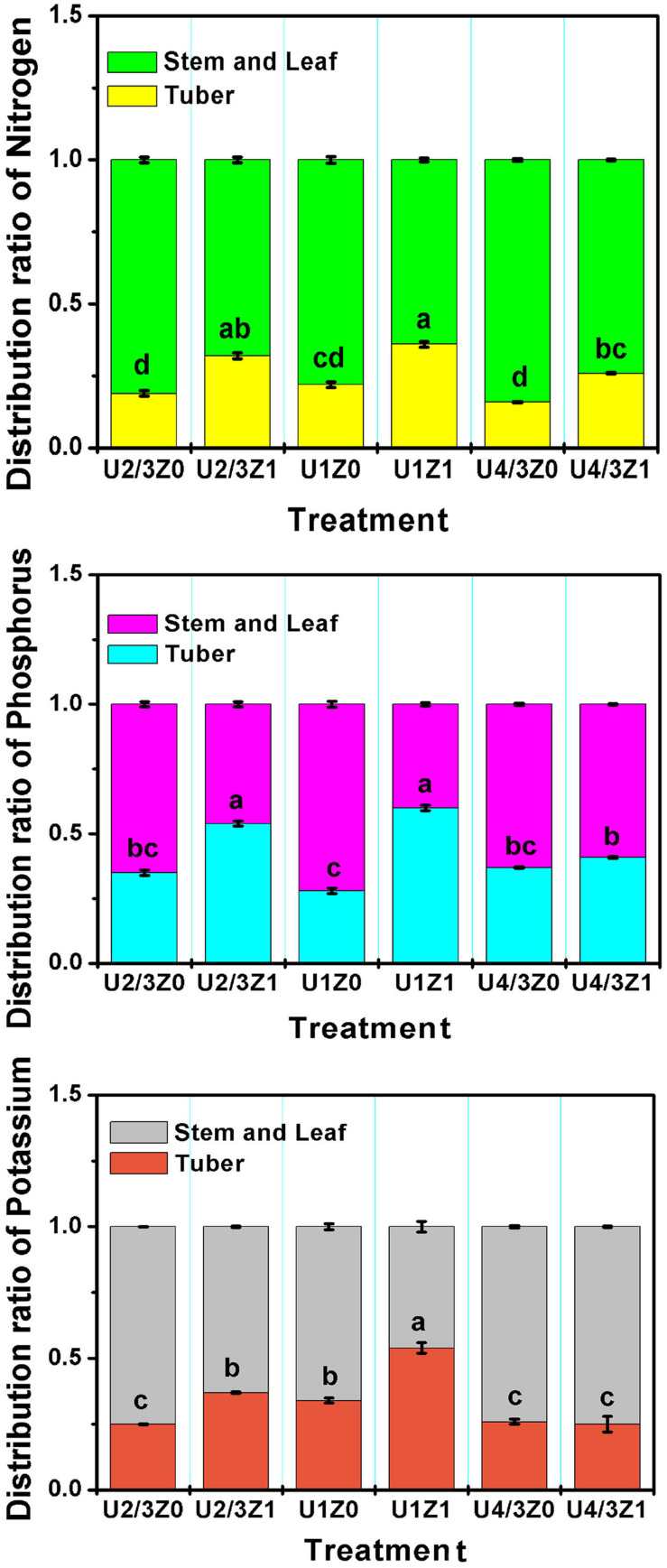
Distribution coefficient of macroelements in potato tubers. *Note:* Means within each column followed by the same letter were not significantly different at the 0.05 level based on one-way ANOVAs followed by Duncan’s multiple range test.

To investigate the bioactivity of ZNC, a field experiment was conducted during one potato growing season. Compared with that of the control treatment (Z_0_), the yield of potato in the ZNC treatment (Z_1_) increased significantly by 4.4–10.4% under the three nitrogen concentrations (Table 4). Compared with Z_0_, Z_1_ significantly improved the distribution coefficients of N, phosphorus and potassium in tubers under the N concentrations of U_2/3_ and U_1_ (Figure 6). Interestingly, soluble protein, starch, soluble sugar, nitrate and V_*C*_ in Z_1_ potatoes were not reduced significantly compared with those in Z_0_ potatoes (Table 4); particularly, soluble protein in U_2/3_Z_1_ was significantly increased compared with that in U_2/3_Z_0_, and the content of Vc in U_1_Z_1_ was significantly increased compared with that in U_1_Z_0_. However, compared with U_4/3_Z_0_, U_4/3_Z_1_ had no significant difference in the distribution coefficients of *P* and K, which resulted in decreases in soluble protein, soluble sugar and nitrate in U_4/3_Z_1_ but increases in yield and starch_._

**TABLE 4:** Quality and yield of fresh potato with different treatments.

Treatment	Soluble protein (mg⋅kg^–^^1^)	Starch (%)	Soluble sugar (%)	Nitrate (g⋅kg^–^^1^)	Vitamin C (mg⋅kg^–^^1^)	Yield (Mg/hm^2^)	Average increment versus control (%)
U_2/3_Z_0_	9.55 c	12.16 b	0.93 b	0.47 bc	13.41 b	11.00 e	-
U_2/3_Z_1_	13.44 a	12.21 b	0.91 b	0.43 c	12.81 b	12.14 d	10.4^1^*
U_1_Z_0_	10.53 bc	12.31 b	1.23 a	0.57 a	14.02 b	13.32 c	-
U_1_Z_1_	11.51 b	12.54 b	1.13 a	0.53 ab	19.94 a	14.10 b	5.8^2^*
U_4/3_Z_0_	14.15 a	10.58 c	1.19 a	0.58 a	17.61 a	14.70 b	-
U_4/3_Z_1_	11.45 b	13.78 a	0.33 c	0.49 bc	21.14 a	15.35 a	4.4^3^*

*Means within each column followed by the same letter were not significantly difference at the 0.05 probability level based on analyses by one-way ANOVAs followed by Duncan’s multiple range test. A dash (-) indicates no data. U_2/3_Z_0_ (100 kg N ha^–1^); U_2/3_Z_1_ (100 kg N ⋅ha^–1^ combined with ZNC); U_1_Z_0_ (150 kg N ⋅ha^–1^); U_1_Z_1_ (150 kg N ⋅ha^–1^ combined with ZNC); U_4/3_Z_0_ (200 kg N ⋅ha^–1^); and U_4/3_Z_1_ (200 kg N ⋅ha^–1^ combined with ZNC).*

^1^*Means average increment versus U_2/3_Z_0_.

^2^*Means average increment versus U_1_Z_0_.

^3^*Means average increment versus U_4/3_Z_0._

## Discussion

The MEs are applied in agriculture instead of living microorganisms, which cannot only reduce ecological risks, but also avoid the problem of unstable activity caused by environmental interference. However, the agricultural research on endophytic metabolites is mostly limited to the laboratory, and the product quality cannot be effectively guaranteed. Therefore, it is of great significance to establish the industrial production and quality control system of MEs (Qin et al., 2018).

Ultrasonic extraction (UAE) was used for the industrial production of MEs, it has multiple advantages over other methods, including a higher extraction rate, shorter extraction time, lower energy consumption and less damage to the product (Chen et al., 2012; Zhang et al., 2014; Tizazu et al., 2018). RSM, as a useful statistical and mathematical method, was used for improving and optimizing processes of industrial production. Special attention should be paid to the results of the 3D response surface and contour lines after the extraction conditions of ZNC were optimized through RSM. Yield of ZNC is significantly positively correlated with the concentration of the added material. However, Wong et al. (2017) found that when the ultrasonic power and time are constant, as the material concentration increases, the yield of ultrasonic extraction will first increase and then decrease significantly. When the maximum output is exceeded, the ultrasonic energy density cannot meet the demand for excessive materials. Amiri et al. (2019) found that, reducing material concentration, enhances the osmotic pressure and contact area, resulting in more solvent being pushed into the cell matrix, and enhancing the penetration of bioactive chemicals through the cell wall into the solvent using ultrasonic-assisted extraction. Before ultrasonic extraction of ZNC, the mycelium was immersed in alcohol for 2 weeks. In the process, the alcohol killed the cells, dissolving or relaxing the cell membrane, which was more conducive to the outflow of cell components, so that the need for energy density was reduced (Keris-Sen et al., 2014). In the ultrasonic extraction process of ZNC, the required energy density is always lower than the supplied energy density, so the yield of ZNC is significantly positively correlated with the material concentration, but has little correlation with the time and power of ultrasonic extraction. Subsequent test results also verified this view. Except for the material ratio, the alcohol concentration is a key factor affecting the extraction of ZNC. This finding is similar to those in cases in which the type and concentration of solvent had significant impacts on ultrasonication-aided lipid extraction from oleaginous microorganisms (Zhang et al., 2014). It was claimed that the solvent selectivity was the most effective parameter on the extent of lipid extraction rather than the bulk convection created by ultrasound (Ranjan et al., 2010). This is because alcohol changes the spatial structure of glycosaminoglycans, proteins and lipids on the fungal cell membrane, thereby fixing channel proteins and changing the fluidity of the cell membrane, which is conducive to the outflow of cell components during the extraction process. At the same time, the dissolved macromolecular chain proteins, polysaccharides, polypeptides and nucleic acids are easily broken into small molecular compounds under ultrasonic conditions. These small molecular compounds are mostly soluble in water, but some compounds have lipid bonds, benzene rings, pyrimidines, carbon chains, and other functional groups cannot be completely soluble in water (Keris-Sen et al., 2014). However, the increase in alcohol concentration in the extract improves the diffusibility and lipophilicity of non-polar compounds, which is conducive to the dissolution of these cell outflow components (Goldstein, 1986). Unlike other production processes that require the addition of chloroform and *n*-butanol (Zhang et al., 2014), the alcohol that added in the extraction process of ZNC has a wider range of sources, lower price, and no peculiar smell. More importantly, the alcohol soaking treatment of mycelium before ultrasound not only avoids ecological risks, it is also more conducive to the extraction of ZNC. In the actual ZNC production process, if X_1_ is greater than 40%, the material is too viscous, which exceeds the capacity of the ultrasonic tank for stirring and discharging. Similarly, the concentration of organic reagents in the tank should be less than 40% according to the ultrasonic extraction tank operation guide. When the concentration of materials and alcohol is unchanged, the extraction time is close to 60 min and the extraction power is close to 6.0 kW, the extraction concentration of ZNC is the largest, which is also consistent with the value calculated by the model, and the prediction of this model is completely credible. Hence, the extraction parameters for maximum production of ZNC were X_1_ was 40.0%, X_2_ was 40.0%, X_3_ was 58.2 min, and X_4_ was 6.0 kW or 12 J L^–1^. Herein, the yield of ZNC in one batch (500 L) met the consumption requirements for field crops in 7,467 hectares (1.125 g⋅ha^–1^ as described previously) (Chen et al., 2020), which proves the feasibility of the UAE and RSM methods for the industrial production of MEs.

The composition and molecular weights of ZNC components were the key factors that affected the HPLC parameters (Nasal et al., 2003). The functional groups in ZNC determined the monitored wavelength, type of chromatographic column and mobile phase, while the molecular weights of ZNC components determined the type of column, limit of detection, retention time and loading amount (Guo et al., 2001). In addition, the antigenicity and immunogenicity of ZNC were also determined by its component composition and molecular weights and were prerequisites for the establishment of ELISA (van de Gaer et al., 2020). The molecular weight distribution of ZNC in this study is different from the results in a previous study (Wang et al., 2020), which may be caused by differences in UAE conditions and equipment. However, the molecular weights and composition of ZNC components obtained from the above results will facilitate the subsequent ZNC evaluation.

The traditional SES-TCMCF method mostly uses C18 series chromatographic columns to directly analyze the fingerprints of samples, which often lacks the systematic study of sample components and the optimization of personalized chromatographic conditions of samples. This method may detect a small number of compounds for a specific sample, and these detected compounds cannot fully represent samples, which causes the problem of low correlation between fingerprint evaluation and sample quality. In this study, compounds such as carbohydrates, amino acids, peptides and nucleic acids together accounted for 96.25% of the total organics in ZNC, and studies have shown that these compounds are effective components and can significantly promote plant growth and disease resistance (Mousa and Raizada, 2013; Vasundhara et al., 2016), which requires key characterization compound of. According to the composition and molecular weight distribution of the ZNC, an amino chromatographic column suitable for these components was used for ZNC HPLC analysis (Filson and Dawson-Andoh, 2009; Li et al., 2012). Whereas, the fingerprints of this method failed to characterize lipid compounds in ZNC, but exogenous lipid compounds that promote plant growth at low concentrations are rarely reported. Therefore, the fingerprints obtained in this study can fully characterize ZNC and the similarity of it is closely related to the stability of ZNC quality. In this study, according to SES-TCMCF of ZNC, the quality of products produced in different batches of large-scale production processes is stable, which may be attributed to the UAE method causing only little damage to ZNC. Because UAE releases intracellular metabolites instantly through cavitation (Clodoveo, 2019). In addition, the alcohol concentration is also a key factor affecting the composition of the compounds in ZNC. The change of alcohol concentration affects the polarity of the extract, and further affects the solubility and composition of carbohydrates, amino acids, peptides and nucleic acids in ZNC (Huffer et al., 2011). Therefore, in order to obtain a uniform quality of ZNC in large-scale production, the concentration of alcohol should be controlled precisely. SES-TCMCF as a simple and efficient method to evaluate unknown compounds in ZNC, provides an effective technical reference for monitoring the production of ZNC to ensure its uniform quality. Moreover, this method lays the foundation for the separation and purification of effective compounds in ZNC by HPLC.

Multiple antibody enzyme-linked immunosorbent assay can detect the specificity of components in ZNC based on the principle of specific binding of antigen and antibody, which is complementary to SES-TCMCF to a certain extent, and avoids the error of sample fingerprint evaluation caused by the similar chromatographic characteristics of the compound (Sun et al., 2009; Gong et al., 2016). Conversely, SES-TCMCF can also avoid false positives and misjudgments common in MA-ELISA (Aydin, 2015). In addition, MA-ELISA is more sensitive, simple, convenient, cheap and easy to popularize in market (Gan and Patel, 2013). When detecting the content of ZNC in other products, there is no need to go through tedious pre-treatment steps like HPLC such as desalination, extraction and purification (van de Gaer et al., 2020), which laying a solid foundation for further detection of ZNC components in fertilizers and pesticides. So, this will be more conducive to the application of ZNC related products.

Accumulating evidence suggests that small molecules such as carbohydrates, proteins, amino acids, and nucleotides are important signals that trigger nutrient metabolism or disease resistance pathways in crops (Carrion et al., 2019; de Vries et al., 2020; Dini-Andreote, 2020; Liu et al., 2020; Oldroyd and Leyser, 2020). Compounds with low molecular weights are not only more easily absorbed by crops but also more stable than high-molecular-weight polymers with similar functions (Li et al., 2010; Han et al., 2014; Peng et al., 2018). ZNC contains such small compounds, and its biological activity is 1,000–100,000-fold higher than that of biostimulants, such as seaweed extracts (Rashidzadeh and Olad, 2014), humic acids (Araujo et al., 2017), and chitosan (Yakhin et al., 2016; Conselvan et al., 2017; Franca et al., 2018). Herein, ZNC significantly increased the yield of field potatoes, which is similar to a 0.6–6.4% increase in maize yield (Chen et al., 2020) and an 8.7–12.1% increase in rice yield (Wang et al., 2020) that have been reported. In addition, ZNC helped transfer macroelements to potato tubers and promoted the transcription of genes such as NRT2.5, NRT1.1, NRT1.5, PHO1 and PHT4; 2 (Lu et al., 2019). Finally, the effect of ZNC on potato yield and quality is significantly affected by the level of N fertilizer application, because N is a more important factor affecting potato growth than ZNC (Oldroyd and Leyser, 2020; Wu et al., 2020). However, it is obvious that at a suitable nitrogen level, industrialized ZNC could increase crop yield without quality reduction, which supports its commercial application in agriculture.

## Conclusion

Ultrasonic extraction was used for industrial production of MEs after its optimization with RSM, and the quality of MEs was further controlled using a novel method including SES-TCMCF, MA-ELISA, and field experiments. The production of ZNC with consistent quality, has been successfully industrialized and applied using these technical systems. Therefore, we established an innovative technical system for the popular application of ZNC, and the technical system is expected to improve agricultural production by requiring less fertilizer and pesticide.

## Data Availability Statement

The original contributions presented in the study are included in the article/supplementary material, further inquiries can be directed to the corresponding author/s.

## Author Contributions

QW and CP analyzed the results and wrote the manuscript under the guidance of MZ, FL and ZL. QW, CP, DZ, LS, HM, and HZ performed the experiments. MZ and FL conceived the idea for the project and wrote the manuscript. All authors read and approved the final manuscript.

## Conflict of Interest

QW, HM, and HZ were employed by the company Shandong Pengbo Biotechnology Co., Ltd. The remaining authors declare that the research was conducted in the absence of any commercial or financial relationships that could be construed as a potential conflict of interest.

## Abbreviations

U _2_/_3_ Z_0_: 100 kg nitrogen ⋅ ha-1
U _2_/_3_ Z_1_: 100 kg nitrogen combined with ZNC ⋅ ha-1
U_1_Z_0_: 150 kg nitrogen ⋅ ha-1
U_1_Z_1_: 150 kg nitrogen combined with ZNC ⋅ ha-1
U _4_/_3_ Z_0_: 200 kg nitrogen ⋅ ha-1
U _4_/_3_ Z_1_: 200 kg nitrogen combined with ZNC ⋅ ha-1
ANOVA: analysis of variance
CCD: central composite design
ESI: electrospray ion source
ELISA: enzyme-linked immunosorbent assay
ZNC: extract from Paecilomyces variotii SJ1
HPLC: high-performance liquid chromatography
MS: mass spectrometer
MEs: metabolites from endophytes
MA-ELISA: multiple antibody enzyme-linked immunosorbent assay
N: Nitrogen
SJ1: Paecilomyces variotii SJ1
PBS: phosphate buffer solution
P: Phosphorus
RSM: response surface methodology
SES-TCMCF: similarity evaluation system of traditional Chinese medicine chromatographic fingerprinting
UAE: ultrasonic extraction.
